# Integrating Fasciolosis Control in the Dry Cow Management: The Effect of Closantel Treatment on Milk Production

**DOI:** 10.1371/journal.pone.0043216

**Published:** 2012-08-20

**Authors:** Johannes Charlier, Miel Hostens, Jos Jacobs, Bonny Van Ranst, Luc Duchateau, Jozef Vercruysse

**Affiliations:** 1 Department of Virology, Parasitology and Immunology, Faculty of Veterinary Medicine, Ghent University, Merelbeke, Belgium; 2 Department of Reproduction, Obstetrics and Herd Health, Faculty of Veterinary Medicine, Ghent University, Merelbeke, Belgium; 3 Elanco Animal Health, Vosselaar, Belgium; 4 Uniform-Agri BV, Assen, The Netherlands; 5 Department of Physiology and Biometrics, Faculty of Veterinary Medicine, Ghent University, Merelbeke, Belgium; Auburn University, United States of America

## Abstract

The liver fluke *Fasciola hepatica* is a parasite of ruminants with a worldwide distribution and an apparent increasing incidence in EU member states. Effective control in dairy cattle is hampered by the lack of flukicides with a zero-withdrawal time for milk, leaving the dry period as the only time that preventive treatment can be applied. Here, we present the results of a blinded, randomized and placebo-controlled trial on 11 dairy herds (402 animals) exposed to *F. hepatica* to 1) assess the effect of closantel treatment at dry-off (or 80–42 days before calving in first-calving heifers) on milk production parameters and 2) evaluate if a number of easy-to-use animal parameters is related to the milk production response after treatment. Closantel treatment resulted in a noticeable decrease of anti-*F. hepatica* antibody levels from 3–6 months after treatment onwards, a higher peak production (1.06 kg) and a slightly higher persistence (9%) of the lactation, resulting in a 305-day milk production increase of 303 kg. No effects of anthelmintic treatment were found on the average protein and fat content of the milk. Milk production responses after treatment were poor in meagre animals and clinically relevant higher milk production responses were observed in first-lactation animals and in cows with a high (0.3–0.5 optical density ratio (ODR)), but not a very high (≥0.5 ODR) *F. hepatica* ELISA result on a milk sample from the previous lactation. We conclude that in dairy herds exposed to *F. hepatica*, flukicide treatment at dry-off is a useful strategy to reduce levels of exposure and increase milk production in the subsequent lactation. Moreover, the results suggest that treatment approaches that only target selected animals within a herd can be developed based on easy-to-use parameters.

## Introduction

The liver fluke *Fasciola hepatica* is a parasite of cattle and sheep with a worldwide distribution. The infection is transmitted by freshwater snails of the family Lymnaeidae. In Europe, the principal intermediate host for *F. hepatica* is the amphibious snail *Galba truncatula*. Under optimal conditions, it takes 6 weeks before an infected snail starts to shed infective cercariae. These cercariae encyst to metacercariae on grass, which are ingested by the final host during grazing. Within the cow, it takes approximately 12 weeks for the parasite to mature to the adult stage and produce eggs that are released via the cow's faeces on pasture [1]. In western Europe, the main period where cattle acquire new infections is the autumn [2].

Because the completion of the life cycle depends on the presence of suitable habitats for the intermediate host, the disease is characterized by a focal distribution [3], [4]. Nonetheless, herd-level prevalences in cattle of 30% to 80% are commonly encountered across Western Europe [5], [6], [7]. Moreover, several studies report increasing incidences of fasciolosis in EU member states and this trend has been primarily attributed to climatic changes, supporting the overwintering of the intermediate host and of the metacercariae [7], [8], [9], [10].

Fasciolosis in cattle is generally subclinical and the negative impact of infections on milk yield is well accepted [11]. Nonetheless, already in 1987 Dargie expressed his concern on the lack of studies to quantify this effect [12]. Very few properly controlled trials to assess the impact of flukicide treatment on milk yield were conducted since then. With the abolition of the E.U. milk quota regulations in 2015, European dairy producers face a future situation with more volatile output prices and competition [13]. In this context, quantification of the production impact of enzootic animal diseases and their control measures is crucial for supporting managerial decisions.

Traditionally, the anthelmintic control of fasciolosis is based on whole-herd treatments with flukicides during the winter period. These treatment schemes have been recommended because most flukicides only have a good activity against the adult stages of *F. hepatica*, which are present during the winter period [14], [15]. However, with the disappearance of flukicides with a zero-withdrawal time for milk in most EU member states, such treatment schemes are no longer economically justifiable and the only period when flukicides can still be administered in lactating animals is during the dry period [16]. To date, no studies are available that evaluate the efficacy or impact on production of flukicide treatment during the dry period. Only few flukicides are registered for use in cows whose milk is used for human consumption, but recently the European Medicines Agency (EMA) recommended a provisional maximum residue limit (MRL) for closantel in milk of bovine and ovine species (EMA/CVMP/846853/2011) to avoid the creation of a therapeutic vacuum.

The objectives of this study were 1) to assess the effect of closantel treatment at dry-off on milk production parameters using a randomized and placebo-controlled study and 2) to evaluate if a number of easy-to-use animal parameters is related to the production response after treatment, allowing a more selective use of flukicides in the future.

## Materials and Methods

### Farm selection criteria

The study was conducted on 12 dairy herds located in Flanders (Belgium). The herds were selected based on the following criteria: (a) herds were naturally exposed to *F. hepatica* based on bulk tank milk ELISA result (≥0.8 optical density ratio (ODR)) indicative for economic losses induced by the infection [6] at the beginning of the trial (September 2010); (b) the animals involved did not receive flukicide treatment ≤6 months before the experimental treatment; (c) application of an average dry period ≥42 days in order to respect the provisional withdrawal period for milk of closantel and (d) storage of herd information and production data in UNIFORM-Agri software (Assen, The Netherlands) enabling standardized collection of the data.

### Study design

This study was a blinded, randomized and controlled clinical trial evaluating the effect of treatment with closantel on milk production parameters and anti-*F. hepatica* antibody levels in milk samples. Animals were drenched with closantel 5% oral solution (Seponver®, Elanco Animal Health) or a placebo (the vehicle liquid of the drug without the active compound) at a dosage of 0.2 ml per kg bodyweight at dry-off. First-calf heifers were drenched between 80 and 42 days before the expected calving date. The active compound and the placebo were dispensed in identical bottles, uniquely labelled with a study and letter code. Nor the farmer, nor the herd veterinarian, nor the principle investigator (JC) knew which letter code corresponded to the active product or placebo. The key was stored at Janssen Animal Health (now Elanco Animal Health) and only revealed after data-analysis.

Treatments (closantel/placebo) were randomly assigned according to Taves' minimization method [17] assuring a proportional assignment of the treatments within herd, infection level (based on pre-treatment *F. hepatica* ELISA result), lactation number and production level.

The study was approved by the Federal Agency for Medicines and Health Products of Belgium, provided a provisional withdrawal time for milk of 42 days was respected (File number 09 VN 1617).

### On farm measurements

The treatment assignments were communicated to the farmer through a hard copy list mentioning cow identification, expected calving date, target date of experimental treatment administration and treatment code. Treatments were performed by the farmer based on estimated bodyweight. At timing of treatment, the farmer was requested to complete the hard copy list with the body condition score (BCS) (according to [18]), estimated bodyweight, administered dose and date of treatment. The progress and compliance to the study was monitored through monthly telephonic contacts and three-monthly farm visits by the principal investigator.

### Collection of milk samples and *Fasciola hepatica* milk ELISA

Bulk tank milk samples were collected with monthly intervals from the beginning of the study (September 2010) until July 2011. Individual milk samples from all lactating animals were collected with three-monthly intervals from July 2010 onwards. The samplings occurred as part of the routine samplings for quality control and milk production registration programmes in cooperation with Milk Control Centre Flanders (Lier, Belgium) and CRV (Arnhem, The Netherlands). The samples were immediately kept on ice during transport and stored at −20°C in the laboratory until analysis.

The collected milk samples were subjected to a *F. hepatica* ELISA as previously described [19]. This ELISA quantifies IgG antibodies binding to the excretory-secretory products of *F. hepatica* and the test results are expressed as ODR. The ELISA is based on the protocol as described by Salimi-Bejestani et al. [20] who reported a sensitivity and specificity when compared to serum ELISA results of 92% and 88%, respectively. The sensitivity and specificity of the ELISA applied on serum when compared to worm counts was estimated at 87% and 90%, respectively [21]. Animals with an ODR≥0.30 are considered positive for *F. hepatica*. In our study, the aim was to evaluate whether *F. hepatica* ELISA applied on individual milk samples in the previous lactation could be used to identify which animals would benefit most in terms of production responses to anthelmintic treatment.

### Collection of production records and data processing

Production records from the participating herds were extracted from the Dairy Data Warehouse (UNIFORM-Agri, Assen, The Netherlands). The extracted variables were: kg milk, fat concentration (g/kg), protein concentration (g/kg), somatic cell count (SCC)/1000, breed, days in milk and lactation number. Depending on the production registration programme of the farm, milk production records were recorded on a daily, 4-weekly or 6-weekly basis.

Next, the milk production records were subjected to the MilkBot® lactation model (DairySight LLC, Argyle, New York) in order to obtain one aggregate measure of 305 day-milk production per cow lactation. This lactation model is designed for detecting and quantifying effects of disease or management interventions on milk production. The functional form of the MilkBot® lactation model was described by Ehrlich [22]. The model has been previously used to assess the effect of metabolic diseases on milk production [23] and demonstrated higher accuracy and precision than the current dairy herd improvement associations' method for calculating lactation yields in the United States [24]. The model quantifies both the shape and magnitude of lactation curves as a set of parameter values, each of which is associated with a single aspect of lactation curve shape. The parameter “scale” is a measure of magnitude, without changing the shape of the curve. The parameter “ramp” measures the steepness of the post-parturient rise in production. The parameter “decay” is used to measure the rate of decline in production after the peak in milk production. Lactation curve analysis allows detecting changes in the distribution of production that are not apparent when only totals are analyzed.

### Statistical data-analysis

The effect of closantel treatment on anti-*F. hepatica* antibody levels in individual milk samples was investigated by a linear mixed model with herd and cow as random effects and treatment (closantel/placebo), days after treatment and an interaction term between treatment and days after treatment as fixed effects. Because the herds were sampled at 3-monthly intervals, the variable ‘days after treatment’ was categorized in 3 intervals: ‘0–3 months’, ‘3–6 months’, ‘>6 months’.

The effect of closantel treatment on milk production parameters was investigated by a linear mixed model with the production parameter as outcome variable, herd as random effect and treatment (closantel/placebo) as fixed effect. Lactation number (‘1^st^’, ‘2^nd^’, ‘3^rd^ or higher’), breed and the natural logarithm of SCC/1000 were introduced as additional fixed effects because they were considered as potential confounding factors. One herd was excluded from the analysis because no SCC data were available. In addition, two cows with breed Brown Swiss and one with breed Friesian Red and White were removed from the analysis because they all belonged to the closantel group without counter parts in the placebo group. The evaluated production parameters were (predicted) 305 day milk production, the different lactation curve parameters (scale, ramp, decay), average fat content (g/kg), average protein content (g/kg) and Ln(SCC/1000). Cows were only included in the analysis if at least 3 separate production records over a time period of >3 months were available.

The relationship of animal parameters with the production response after treatment was evaluated using the model to estimate the effect of treatment of 305-day milk production. Continuous potential decision parameters (anti-*F. hepatica* antibody level and BCS) were categorized according to their quartiles. If more than one anti-*F. hepatica* antibody measurement was available pre-treatment, the average value was used for categorization. The treatment effect was estimated within each category of the pre-treatment anti-*F. hepatica* antibody level, BCS centred to the herd mean, year quarter of treatment and lactation number.

All statistical analyses were carried out with the PROC MIXED command in the software package SAS version 9.3 (SAS institute Inc., Cary, NC, USA). Normality of the residuals was checked by Q-Q plots. Heteroscedasticity was checked by plots of the residuals vs. predicted values. Additional diagnostic plots were performed to assess independence of the residuals and linearity of the means. Variance component estimation was based on restricted maximum likelihood and statistical significance of fixed effects was based on F-statistics.

## Results

### Herd characteristics before treatment and treatment allocation

Treatment records and ELISA results to analyse the effect of treatment on anti-*F. hepatica* antibody levels were available for 475 cows from 12 herds. The closantel and placebo group in this analysis consisted of 246 and 229 animals, respectively. The average anti-*F. hepatica* antibody level in individual milk samples in the period before treatment administration was similar in both treatment groups (Table 1).

**Table 1:** Number of cows, average ± standard deviation of anti-*F. hepatica* antibody levels before treatment and 305-day milk production in the lactation before treatment and distribution of breed, parity and year quarter of treatment in the 2 treatment groups.

Parameter	Closantel	Placebo
N° of cows	208	194
Anti-*F. hepatica* antibody level (ODR)	0.36±0.26	0.40±0.27
305-day milk production (kg)	9,012±1,661	9,059±1,742
Breed:		
Holstein Friesian	45.3	43.0
Dutch Friesian	6.5	5.2
Parity (%):		
1st	11.2	10.5
2nd	16.7	13.7
≥3rd	23.9	24.1
Year quarter:		
Jan–Mar	12.4	11.9
Apr–Jun	5.2	4.7
Jul–Sep	16.9	15.7
Oct–Dec	17.2	15.9

The analysis of the effect of treatment on milk production parameters was based on data from 402 cows from 11 herds. Table 1 shows the characteristics of the cows included in the analysis of the effect of treatment on milk production parameters: the 305-day milk production in the period before treatment was similar and the number of cows in the treatment groups, parity, breed and year quarter in which the treatment was performed was evenly distributed between the 2 treatment groups, indicating a successful treatment allocation.

### Anti-*Fasciola hepatica* antibody levels in milk

**Figure 1:**
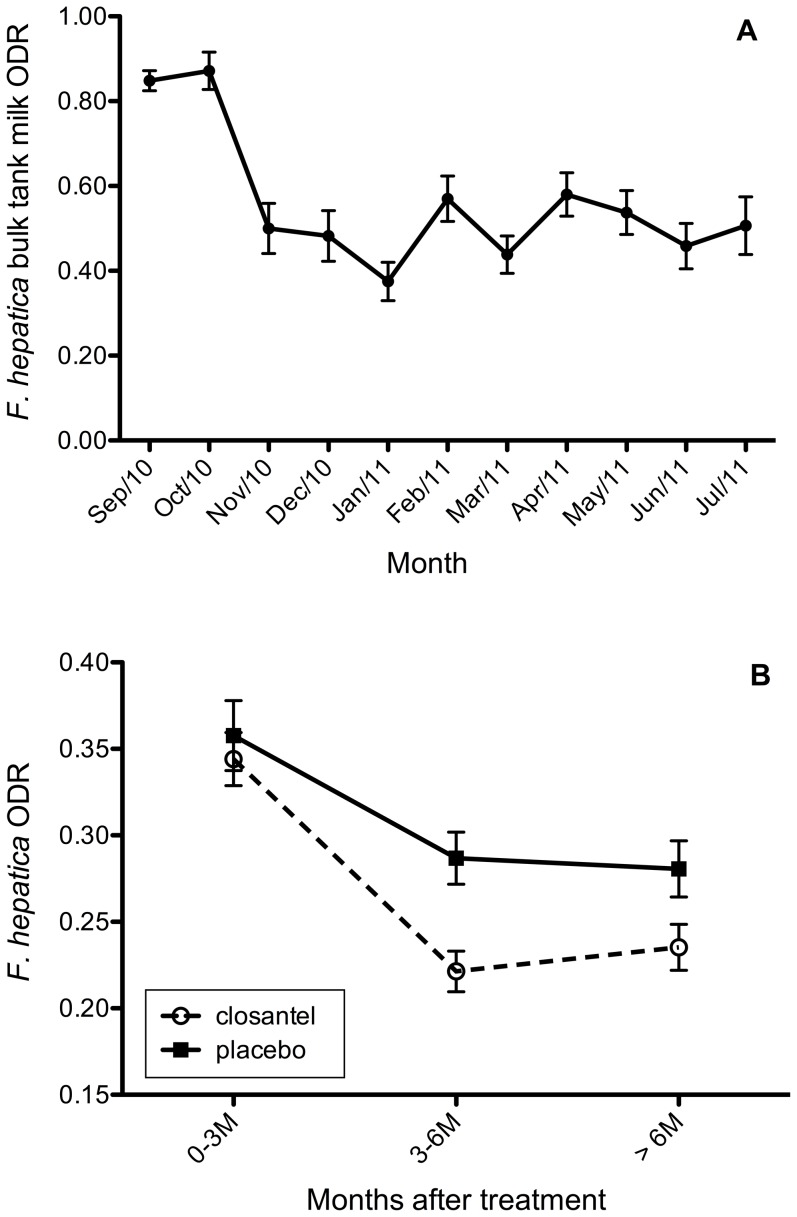
The course of anti-*F. hepatica* antibody levels (ODR) during the study period in bulk-tank milk samples from the 12 studied herds (A) and relative to the months after experimental treatment in individual milk samples of 475 cows in the 12 herds (B). Bars represent standard error of the mean.

The course of anti-*F. hepatica* antibody levels in bulk-tank milk during the study period is shown in Fig. 1A. Two months after the start of the trial, the mean bulk-tank milk antibody level showed a substantial decrease and subsequently remained at a more or less stable level. The course of anti-*F. hepatica* antibody levels in individual milk samples relative to the time post-treatment is shown Fig. 1B. The anti-*F. hepatica* antibody levels decreased after treatment in both the closantel and the placebo-group (*P*<0.001) with significantly lower antibody levels in the closantel group than in the placebo group (*P* = 0.05). The interaction term between treatment and days after treatment was not significant (*P* = 0.11). The proportion of the total variation in anti-*F. hepatica* antibody levels that resided at the herd, cow and residual level was 7, 51 and 42% respectively, indicating that there was a great variation in anti-*F. hepatica* antibody levels between cows within a herd.

### Overall treatment effect on milk production

**Figure 2:**
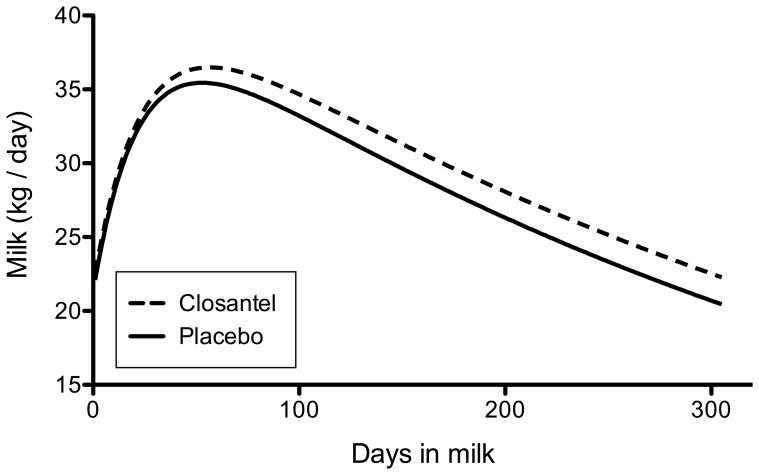
The average lactation curve of cows following treatment with closantel or a placebo at dry-off (Curves represent the data for Holstein Friesian cows in ≥3^rd^ lactation).

The results of the linear mixed model to evaluate the effect of closantel treatment on 305-day milk production are given in Table 2. After controlling for the factors lactation number, breed and somatic cell count, the 305-day milk production in the closantel group was increased by 303 kg (*P* = 0.026), which corresponds to a milk production response of 0.99 kg/day per cow. No significant effects of anthelmintic treatment were found on the average protein (*P* = 0.93) and fat content (*P* = 0.58) of the milk produced or on somatic cell counts (*P* = 0.45). Least square means of these variables in both treatment groups are given in Table 3. Finally, the MilkBot® parameters (scale, ramp, decay) in Table 3 and the resulting lactation curve in Fig. 2 show how the shape of the lactation curve was modified by treatment. The results suggest that closantel treatment resulted in a higher peak production and a slightly higher persistence (9%) of the lactation.

**Table 2:** The results of a linear mixed model to estimate the effect of closantel treatment at dry-off (or approximately 42 days before calving for heifers) on 305-day milk production in 11 herds exposed to *F. hepatica* (based on 402 cows).

Variable	*β*	S.E.	*P*
Intercept	10,685	544	<0.001
Closantel (vs. placebo)	303	135	0.026
Lactation number (baseline = 3^rd^ and higher):			<0.001
First	−2,522	189	
Second	−543	163	
Breed (baseline = Holstein Friesian):			
Dutch Friesian	−256	259	0.324
Ln (SCC^a^/1000)	−158	61	0.009

Random effects	Variance	S.E.	Proportion of total variance (%)
Herd	2,007,621	924,635	53
Residual	1,812,943	130,498	47

a Somatic cell count.

**Table 3:** Least square means^a^ (standard error of the mean) of average protein and fat concentration and milk production parameters following treatment in the closantel and placebo group.

Variable	Closantel	Placebo	*P*
Average protein content (g/kg)	31.8 (0.6)	31.8 (0.6)	0.93
Average fat content (g/kg)	38.7 (0.9)	38.4 (0.9)	0.58
Ln (SCC^b^/1000)	5.05 (0.15)	4.96 (0.15)	0.45
Scale	43.59 (2.26)	42.53 (2.27)	0.14
Ramp	24.98 (0.89)	23.69 (0.91)	0.05
Decay	0.0022 (0.00009)	0.0024 (0.00009)	0.10

*P*-values evaluate the difference between the 2 treatment groups.

a Least square means for Holstein Friesian Cows in ≥3^rd^ lactation.

b Somatic cell count.

### The relationship between animal parameters and the milk yield response after anthelmintic treatment

**Figure 3:**
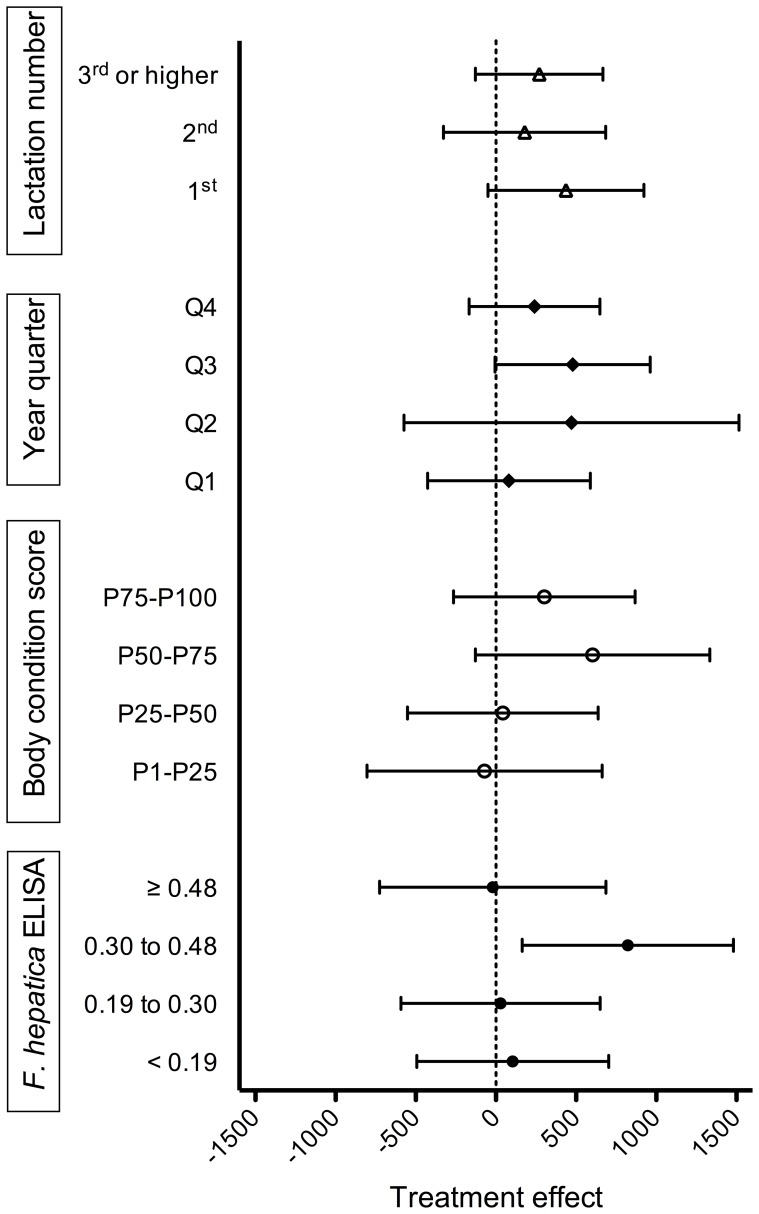
The estimated effect of closantel treatment at dry-off (or approximately 42 days before calving for heifers) on 305-day milk production according to several potential decision parameters for selective anthelmintic treatment. Error bars represent the 95%-confidence interval of the treatment-effect. Categories for *F. hepatica* ELISA results and body condition score represent quartiles.

The effect of closantel treatment on milk production according to several potential decision parameters is given in Fig. 3. The highest treatment effect was found in cows with a pre-treatment *F. hepatica* ELISA result between 0.30 and 0.48 ODR (3^rd^ quartile). In this category, the 305-day milk production was increased by 823 kg (95% confidence interval: 164, 1482). No treatment effect was observed in the highest *F. hepatica* ODR category (4^th^ quartile). Other potential trends were greater milk production responses after closantel treatment in cows with increasing BCS, in cows treated during the third year quarter and in first-lactation cows. However, the 95%-confidence intervals of these categories all included 0.

## Discussion

To our knowledge, this is the first blinded, randomized placebo-controlled study on the effect of fasciolicide treatment on milk yield. Previous studies only investigated associations between *F. hepatica* infection status and milk yield [19], [25], or assessed the effect of chemotherapy but lacked a placebo-administered control group, randomization and follow-up during a whole lactation [26], [27], [28]. The anthelmintic treatments and the recording of potential decision parameters were performed by the farmers, therefore the results of this study are considered repeatable under field circumstances.

Previously, we showed that an increase in the anti-*F. hepatica* level in bulk tank milk over the interquartile range was associated with a drop in the annual average milk yield of 3% [19]. Here, we show that these losses are to a large extent recoverable by anthelmintic treatment. Closantel treatment at dry-off resulted in a 3.3% increase in milk yield (1 kg/cow per day) in the subsequent lactation. This effect was mainly due to a better start-off of the lactation with a steeper rise in production and a higher peak production, suggesting it is induced by an improved liver metabolism post-partum. The observed effect of 3.3% increase in milk yield is considerably lower than in previous studies where milk production responses up to 8 and 15% after flukicide treatment are reported [26], [27]. However, these authors failed to monitor milk production over a whole lactation and such production responses may thus be realistic on a short term or in individual animals as observed here in animals with a *F. hepatica* ODR between 0.3 and 0.5, but not on an average basis. Nonetheless, compared with the cost of an anthelmintic dose, a treatment response of 3.3% increase in milk yield represents approximately a 10-fold return on investment [29].

In contrast to previous studies, we could not observe an effect of flukicide treatment on the fat content of the milk [28], [30]. Because we investigated the effect of treatment on the average fat concentration over the whole lactation, such effect may still have been present on a short term after treatment, which was not detectable in our analysis.

We must recognize that probably not all of the observed effects on milk yield can be ascribed to reduction in the *F. hepatica* burden. Closantel is also active against other blood-feeding parasites of cattle such as *Haemonchus* spp. and *Hypoderma bovis* [31]. However, the prevalence of these parasites in dairy cows in the study area is low [32], [33], [34]. Moreover, the significant drop in anti-*F. hepatica* levels post-treatment suggests that the majority of the effect of closantel treatment on milk yield is caused by its activity against *F. hepatica*.

As reported in other studies [35], [36], the difference in anti-*F. hepatica* antibody level between the 2 treatment groups could only be clearly observed >3 months post-treatment. The significant drop in anti-*F. hepatica* antibody levels suggests that treatment at dry-off resulted in a successful reduction of the worm burden, even if these treatments were applied throughout the year when immature stages of *F. hepatica* are present. Closantel has only a partial activity against immature stages (<9 weeks old) of *F. hepatica* in the cow [15], [37]. However, in our study the population of immature stages may have been small. This is suggested by the decrease in anti-*F. hepatica* antibody levels that was observed in the placebo-treated animals and in the bulk-tank milk during the study. Low levels of reinfection may be attributed to the treatment of part of the herd and the climatic conditions that were considered unfavourable for the development of free-living stages of *F. hepatica* during the study period (2010–2011) (Based on the reports of the Prognosis Commission on liver fluke of the Netherlands; L. Moll, personal communication).

It is of major concern to food safety organisations and dairy cooperatives to prevent the presence of medicine residues above the MRL in commercialized milk. Selective use of flukicides will therefore become of increasing importance to reduce the consumer's risk of exposure to residues in milk. Moreover, selective use of anthelmintics is considered to reduce the development of anthelmintic resistance [38]. In this context, treatment at dry-off where only few individuals are treated at a given moment is a safer strategy than the traditional whole-herd treatments. Moreover, our results suggest that it is possible to identify animals within a herd that will benefit most from anthelmintic treatment by easy-to use animal parameters, thus enabling a more selective use of anthelmintics. The animal parameters were evaluated based on a randomized study. Therefore, observed differences are likely to have a causal relationship with the treatment.

Clinically relevant higher production responses after treatment were observed in animals with a pre-treatment ODR between 0.3 to 0.5, in animals with a higher BCS at dry-off (3^rd^ quartile), in first-lactating animals and treatments administered in the 3^rd^ year quarter. Higher treatment responses in the 3^rd^ year quarter may be explained by the fact that this was in the beginning of the trial when the levels of infection were considered highest (see discussion above). Higher treatment responses in animals with a pre-treatment ODR >0.3 could be expected because animals above this threshold are considered infected with *F. hepatica*. However, the lack of milk yield response after treatment in the highest pre-treatment ODR category (0.5–1.5) is striking. There is no reason to believe that this is related to lack of anthelmintic efficacy. However, it has been shown that *F. hepatica* ODR is correlated with infection intensity and extent of liver pathology [21], [39], [40] suggesting that remaining liver pathology after treatment prevented the occurrence of a clear production response in these animals. This was also suggested as the reason for lack of production response in retired dairy cows where closantel treatment efficiently removed the worm burden, but did not induce increased weight gain [41]. Alternatively, lack of production responses in very high-ODR animals could also be caused by a-specific reactions in the *F. hepatica* ELISA such as those that can occur in milk samples from mastitis-affected udders [42]. The observed relationship between BCS and milk yield response after treatment appears highly similar as the documented quadratic relationship between BCS around partus and subsequent milk production with an optimum milk production in cows with a BCS (on a 5-point scale) of 3.5 [43]. In our study poor production responses were observed in animals in the first and second quartile of BCS and highest production responses in animals in the third quartile of BCS. The greater milk yield with increasing BCS to an optimum is considered to be the result of a greater availability of energy for the cow. By comparison, the reduction in milk yield when the optimum calving BCS is surpassed is considered as the result of lower dry-matter intake in overconditioned cows [43]. BCS may thus be an important parameter for selective flukicide treatment, but our study did not look into potential weight gain responses after treatment in low BCS animals. Finally, the higher treatment responses in 1^st^ lactation animals can be explained by the typical different quality of pastures grazed by heifers compared to milking cows. The better pastures are typically preserved for the milking cows; heifer pastures being more humid and displaying more vegetation diversity thus exhibiting a greater transmission potential for *F. hepatica* infection [4], [44].

In conclusion, in this randomized controlled field study, we demonstrated that in dairy herds exposed to *F. hepatica*, closantel treatment at dry-off is a useful strategy to reduce the levels of infection with *F. hepatica* and increase milk production in the subsequent lactation. Production responses were highest in first-lactating animals and in animals with a high (0.3–0.5 ODR), but not very high (>0.5 ODR) anti-*F. hepatica* antibody level in pre-treatment milk samples, while they remained poor in meagre animals. We propose to use age and anti-*F. hepatica* antibody level in pre-treatment milk samples as easy-to-use animal parameters for selective treatment within a herd. This will likely reduce the risk of unwanted flukicide residues in milk. Further research is required to assess the economic impact of our findings and of selective treatment approaches.

## References

[1] Andrews SJ (1999) The life cycle of *Fasciola hepatica*. In: Dalton JP editor. Fasciolosis. Oxon: CABI Publishing. pp. 1–20.

[2] Gaasenbeek CPH, Over HJ, Noorman N, de Leeuw WA (1992) An epidemiological study of *Fasciola hepatica* in The Netherlands. Vet Quart 14: 140–144. 10.1080/01652176.1992.9694351 1485403

[3] Zukowksi SH, Wilkerson GW, Malone JB (1993) Fasciolosis in cattle in Louisiana. II. Development of a system to use soil maps in a geographic information system to estimate disease risk on Louisiana coastal marsh rangeland. Vet Parasitol 47: 51–65. 8493767 10.1016/0304-4017(93)90175-m

[4] Charlier J, Bennema SC, Caron Y, Counotte M, Ducheyne E, et al (2011) Towards assessing fine-scale indicators for the spatial transmission risk of *Fasciola hepatica* in cattle. Geospatial Health 5: 239–245. 21590674 10.4081/gh.2011.176

[5] Mezo M, González-Warleta M, Castro-Hermida JA, Ubeira FM (2008) Evaluation of the flukicide treatment policy for dairy cattle in Galicia (NW Spain). Vet Parasitol 157: 235–243. 18774648 10.1016/j.vetpar.2008.07.032

[6] Bennema S, Vercruysse J, Claerebout E, Schnieder T, Strube C, et al (2009) The use of bulk-tank milk ELISAs to assess the spatial distribution of *Fasciola hepatica*, *Ostertagia ostertagi* and *Dictyocaulus viviparus* in dairy cattle in Flanders (Belgium). Vet Parasitol 165: 51–57. 19656630 10.1016/j.vetpar.2009.07.006

[7] McCann CM, Baylis M, Williams DJL (2010) Seroprevalence and spatial distribution of *Fasciola hepatica*-infected dairy herds in England and Wales. Vet Rec 166: 612–617. 20472872 10.1136/vr.b4836

[8] van Dijk J, Sargison ND, Kenyon F, Skuce PJ (2010) Climate change and infectious disease: helminthological challenges to farmed ruminants in temperate regions. Animal 3: 377–392. 10.1017/S1751731109990991 22443942

[9] Fox NJ, White PCL, McClean CJ, Marion G, Evans A, et al (2011) Predicting impacts of climate change on *Fasciola hepatica* risk. Plos One e16126. 21249228 10.1371/journal.pone.0016126 PMC3018428

[10] Fairweather I (2011) Reducing the future threat from (liver) fluke: realistic prospect or quixotic fantasy? Vet Parasitol 180: 133–14. 21703766 10.1016/j.vetpar.2011.05.034

[11] Torgerson P, Claxton J (1999) Epidemiology and Control. In: Dalton JP editor. Fasciolosis. Oxon: CABI Publishing. pp. 113–149.

[12] Dargie JD (1987) The impact on production and mechanisms of pathogenesis of trematode infections in cattle and sheep. Int J Parasitol 17: 453–463. 3294657 10.1016/0020-7519(87)90121-4

[13] Jongeneel RC, van Berkum S, de Bont C, van Bruchem C, Helming J, et al.. (2010) European dairy policy in the years to come: Quota abolition and competitiveness. LEI, The Hague, Report N°. 2010-017.

[14] Bossaert K, Lonneux J-F, Losson B, Peeters J (1999) Fasciolosis incidence forecasts in Belgium by means of climatic data. Ann Méd Vét 143: 201–210.

[15] Fairweather I, Boray JC (1999) Fasciolicides: efficacy, actions, resistance and its management. Vet J 158: 81–112. 10489266 10.1053/tvjl.1999.0377

[16] O' Brien B, Jordan K, Danaher M (2010) Update on the use of flukicides. Irish Vet J 63: 702–704.

[17] Staquet M, Dalesio O (1990) Designs for phase III trials. In: Buyse ME, Staquet MJ, Sylvester RJ editors. Cancer Clinical Trials Methods and Practice. Oxford: Oxford University Press. pp. 260–275.

[18] Edmonson AJ, Lean IJ, Weaver LD, Farver T, Webster G (1989) A body condition scoring chart for holstein dairy-cows. J Dairy Sci 72: 68–78.

[19] Charlier J, Duchateau L, Claerebout E, Williams D, Vercruysse J (2007) Associations between anti-*Fasciola hepatica* antibody levels in bulk-tank milk samples and production parameters in dairy herds. Prev Vet Med 78: 57–66. 17095109 10.1016/j.prevetmed.2006.09.010

[20] Salimi-Bejestani MR, Daniel R, Cripps P, Felstead S, Williams DJL (2007) Evaluation of an enzyme-linked immunosorbent assay for detection of antibodies to *Fasciola hepatica* in milk. Vet Parasitol 149: 290–293. 17826912 10.1016/j.vetpar.2007.08.008

[21] Charlier J, De Meulemeester L, Claerebout E, Williams D, Vercruysse J (2008) Qualitative and quantitative evaluation of coprological and serological techniques for the diagnosis of fasciolosis in cattle. Vet Parasitol 153: 44–51. 18329811 10.1016/j.vetpar.2008.01.035

[22] Ehrlich J (2011) Quantifying shape of lactation curves, and benchmark curves for common dairy breeds and parities. Bovine Practioner 45: 88–95.

[23] Hostens M, Ehrlich J, Van Ranst B, Opsomer G (2012) On farm evaluation of the effect of metabolic diseases on the shape of the lactation curve in dairy cows through the Milkbot lactation model. J Dairy Sci 95: 2988–3007. 22612936 10.3168/jds.2011-4791

[24] Cole JB, Ehrlich JL, Null DJ (2012) Projecting milk yield using best prediction and the milkbot lactation model. J Dairy Sci 95: 4041–4044. 22720958 10.3168/jds.2011-4905

[25] Mezo M, González-Warleta M, Castro-Hermida JA, Muiño L, Ubeira FM (2011) Association between anti-*F. hepatica* antibody levels in milk and production losses in dairy cows. Vet Parasitol 180: 237–242. 21459514 10.1016/j.vetpar.2011.03.009

[26] Ross JG (1970) The economics of *Fasciola hepatica* infections in cattle. Br Vet J 126: 13–15. 5464998

[27] Randell WF, Bradley RE (1980) Effects of hexachlorethane on the milk yields of dairy cows in North Florida infected with *Fasciola hepatica* . Am J Vet Res 41: 262–263. 7369597

[28] Khan MK, Sajid MS, Khan MN, Iqbal Z, Arshad M, et al (2011) Point prevalence of bovine fascioliasis and the influence of chemotherapy on the milk yield in a lactating bovine population from the district of Toba Tek Singh, Pakistan. J Helminthol 85: 334–338. 21062526 10.1017/S0022149X10000659

[29] Charlier J, Van der Voort M, Hogeveen H, Vercruysse J (2012) ParaCalc® - a novel tool to estimate the costs of worm infections on the dairy herd. Vet Parasitol 184: 204–211. 21978741 10.1016/j.vetpar.2011.09.008

[30] Black NM, Froyd G (1972) The possible influence of liver fluke infestation on milk quality. Vet Rec 90: 71–72. 5074105 10.1136/vr.90.3.71

[31] Guerrero J (1984) Closantel: a review of its antiparasitic activity. Prev Vet Med 2: 317–327.

[32] Agneessens J, Claerebout E, Dorny P, Borgsteede FHM, Vercruysse J (2000) Nematode parasitism in adult dairy cows in Belgium. Vet Parasitol 90: 83–92. 10828514 10.1016/s0304-4017(00)00232-6

[33] Borgsteede FHM, Tibben J, Cornelissen JBWJ, Agneessens J, Gaasenbeek CPH (2000) Nematode parasites of adult dairy cattle in the Netherlands. Vet Parasitol 89: 287–296. 10799842 10.1016/s0304-4017(00)00219-3

[34] Haine D, Boelaert F, Pfeiffer DU, Saegerman C, Lonneux J-F, et al (2004) Herd-level seroprevalence and risk-mapping of bovine hypodermosis in Belgian cattle herds. Prev Vet Med 65: 93–104. 15454329 10.1016/j.prevetmed.2004.06.005

[35] Levieux D, Levieux A, Mage C, Garel J-P (1992) Immunological detection of chemotherapeutic success in bovine fasciolosis using the specific antigen f2. Vet Parasitol 45: 81–88. 1485423 10.1016/0304-4017(92)90029-9

[36] Boulard C, Carreras F, Van Gool F (1995) Evaluation of nitroxynil and closantel activity using ELISA and egg counts against *Fasciola hepatica* in experimentally and naturally infected cattle. Vet Res 26: 249–255. 7550396

[37] Hanna REB, Cromie L, Taylor SM, Couper A (2006) The effect of a parenteral ivermectin/closantel injection on the growth and reproductive development of early immature *Fasciola hepatica* in cattle. Vet Parasitol 142: 79–90. 10.1016/j.vetpar.2006.06.025 16901648

[38] van Wyk JA, Hoste H, Kaplan RM, Besier RB (2006) Targeted selective treatment for worm management – How do we sell rational programs to farmers? Vet Parasitol 139: 336–346. 16774807 10.1016/j.vetpar.2006.04.023

[39] Marcos LA, Yi P, Machicado A, Andrade R, Samalvides F, et al (2007) Hepatic fibrosis and *Fasciola hepatica* in cattle. J Helminthol 81: 381–386. 17958928 10.1017/S0022149X07850231

[40] Salimi-Bejestani MR, Cripps P, Williams DJL (2008) Evaluation of an ELISA to assess the intensity of *Fasciola hepatica* infection in cattle. Vet Rec 162: 109–111. 18223266 10.1136/vr.162.4.109

[41] Mage C, Levieux D, Bernabe P, Degez P (1993) Liver fluke therapy by closantel in culled dairy cows. Revue Méd Vét 144: 425–429.

[42] Charlier J, Duchateau L, Vangroenweghe F, Claerebout E, Burvenich C, et al (2006) The effect of an experimentally induced acute mastitis on the test results of an *Ostertagia ostertagi* ELISA. Vet Parasitol 136: 161–165. 16300898 10.1016/j.vetpar.2005.10.017

[43] Roche JR, Friggens NC, Kay JK, Fisher MW, Stafford KJ, et al (2009) Invited review: Body condition score and its association with dairy cow productivity, health, and welfare. J Dairy Sci 92: 5769–5801. 19923585 10.3168/jds.2009-2431

[44] Rondelaud D, Hourdin P, Vignoles P, Dreyfuss G, Cabaret J (2011) The detection of snail host habitats in liver fluke infected farms by use of plant indicators. Vet Parasitol 181: 166–173. 21524858 10.1016/j.vetpar.2011.03.056

